# Effects of hesperidin in orange juice on blood and pulse pressures in mildly hypertensive individuals: a randomized controlled trial (Citrus study)

**DOI:** 10.1007/s00394-020-02279-0

**Published:** 2020-07-13

**Authors:** Rosa M. Valls, Anna Pedret, Lorena Calderón-Pérez, Elisabet Llauradó, Laura Pla-Pagà, Judit Companys, Ana Moragas, Francisco Martín-Luján, Yolanda Ortega, Montse Giralt, Marta Romeu, Laura Rubió, Jordi Mayneris-Perxachs, Núria Canela, Francesc Puiggrós, Antoni Caimari, Josep M. Del Bas, Lluís Arola, Rosa Solà

**Affiliations:** 1 Functional Nutrition, Oxidation, and Cardiovascular Diseases Group (NFOC-Salut), Universitat Rovira i Virgili, Facultat de Medicina i Ciències de La Salut, Reus, Spain; 2 Eurecat, Centre Tecnològic de Catalunya, Unitat de Nutrició i Salut, Av. de La Universitat, 1, 43204 Reus, Spain; 3 Departament de Medicina i Cirurgia, Universitat Rovira i Virgili, Reus, Spain; 4 Institut Universitari d’Investigació en Atenció Primària-IDIAP Jordi Gol, Tarragona, Spain; 5 Primary Care Centre Jaume I, Institut Català de la Salut, Tarragona, Spain; 6 Primary Care Centre El Morell, Institut Català de la Salut, Tarragona, Spain; 7 Primary Care Centre Salou, Institut Català de la Salut, Tarragona, Spain; 8 Eurecat, Centre Tecnològic de Catalunya, Centre for Omic Sciences, Reus, Spain; 9 Departament de Bioquímica i Biotecnologia, Grup de Recerca en Nutrigenòmica, Universitat Rovira i Virgili, Tarragona, Spain; 10 Hospital Universitari Sant Joan de Reus, Reus, Spain; 11 Present Address: Food Technology Department, XaRTA-TPV, Agrotecnio Center, Escola Tècnica Superior d’Enginyeria Agrària, University of Lleida, Av/ Alcalde Rovira Roure 191, 25198 Lleida, Spain

**Keywords:** Orange juice, Hesperidin, Blood pressure, Pulse pressure, Pre-hypertension

## Abstract

**Purpose:**

To assess the sustained and acute effects, as well as the influence of sustained consumption on the acute effects, of orange juice (OJ) with a natural hesperidin content and hesperidin-enriched OJ (EOJ) on blood (BP) and pulse (PP) pressures in pre- and stage-1 hypertensive individuals.

**Methods:**

In a randomized, parallel, double-blind, placebo-controlled trial, participants (*n* = 159) received 500 mL/day of control drink, OJ, or EOJ for 12 weeks. Two dose–response studies were performed at baseline and after 12 weeks.

**Results:**

A single EOJ dose (500 mL) reduced systolic BP (SBP) and PP, with greater changes after sustained treatment where a decrease in diastolic BP (DBP) also occurred (*P* < 0.05). SBP and PP decreased in a dose-dependent manner relative to the hesperidin content of the beverages throughout the 12 weeks (*P* < 0.05). OJ and EOJ decreased homocysteine levels at 12 weeks versus the control drink (*P* < 0.05). After 12 weeks of EOJ consumption, four genes related to hypertension (PTX3, NLRP3, NPSR1 and NAMPT) were differentially expressed in peripheral blood mononuclear cells (*P* < 0.05).

**Conclusion:**

Hesperidin in OJ reduces SBP and PP after sustained consumption, and after a single dose, the chronic consumption of EOJ enhances its postprandial effect. Decreases in systemic and transcriptomic biomarkers were concomitant with BP and PP changes. EOJ could be a useful co-adjuvant tool for BP and PP management in pre- and stage-1 hypertensive individuals.

**Electronic supplementary material:**

The online version of this article (10.1007/s00394-020-02279-0) contains supplementary material, which is available to authorized users.

## Introduction

Flavonoid compounds are the most abundant phenolic compounds in plants, and citrus flavonoids, particularly present in orange juice (OJ), are attracting attention due to their beneficial effects on cardiovascular risk factors [1].

OJ is a main dietary source of flavanones, a subclass of flavonoids, and hesperetin-7-*O*-rutinoside (hesperidin) and naringenin-7-*O*-rutinoside (narirutin) are the main citrus flavanone components [2].

Data from cohort studies reported an inverse association between citrus fruit/flavanone consumption and cerebrovascular disease [3–5] and cardiovascular mortality [6–8]. Antihypertensive, antithrombotic, anti-inflammatory, antilipemic, vasodilator, and antioxidant effects of hesperidin have been reported in animal models [9–11]. Similar outcomes have been observed for narirutin [12].

Recently, hesperidin has been shown to reduce the atherosclerotic plaque area and macrophage foam cell formation in low density lipoprotein (LDL) receptor-deficient mice [11]. The aforementioned properties of hesperidin have been considered to be the mechanisms responsible for the beneficial effects of citrus flavanone consumption on cardiovascular disease in humans [13].

Concerning the antihypertensive effects of citrus flavanones, data from animal studies showed that hesperetin, a biological metabolite of hesperidin [10], exerts an antihypertensive effect in hypertensive rats but not in normotensive rats [9, 14]. The antihypertensive effect, as well as vasodilatory and anti-inflammatory activities, has been reported to be mediated by the hesperetin-7-*O*-β-d-glucuronide conjugate [10]. In humans, a natural OJ, but not a hesperidin-enriched beverage, decreased blood pressure (BP) in overweight and obese individuals [15]. Similarly, chronic consumption of hesperidin reduced BP in type 2 diabetes patients [16], although no hypotensive effect was observed in healthy or overweight individuals [17, 18]. In individuals at moderate risk of cardiovascular disease (CVD), no changes in BP or other cardiovascular risk biomarkers were observed after a single dose of OJ or a hesperidin supplement at 5 h post intake [19]. Among flavonoids subclasses, flavone and flavan-3-ol compounds, but not flavanones, were related to the prevention of hypertension in a cohort of 87,242 women from the Nurses’ Health Study [20].

Thus, data of the antihypertensive effect of hesperidin consumption in humans remain controversial. Therefore, we assessed both the sustained and acute effects, as well as the influence of sustained consumption on acute effects, of real-life doses of OJ and a hesperidin-enriched dose on BP, pulse pressure (PP), and cardiovascular risk biomarkers in pre- and stage-1 hypertensive individuals. Our hypothesis was that hesperidin in OJ would provide benefits on BP and PP not only after sustained consumption but also at postprandial level after a single dose.

## Materials and methods

### Study population

Participants from the general population were recruited by means of news in the newspapers, social networks, and tableaux advertisements in the Hospital Universitari Sant Joan (HUSJ)-Eurecat, Reus, Spain, between January 2016 and June 2017. From 311 subjects assessed for eligibility, 159 (53 women and 106 men) pre- or stage-1 hypertensive individuals, according to current guidelines [21], were recruited. Inclusion criteria were as follows: age from 18 to 65, systolic blood pressure (SBP) ≥ 120 mmHg, no family history of cardiovascular disease or chronic disease, and willingness to provide informed consent before the initial screening visit. Exclusion criteria were: body mass index (BMI) ≥ 35 kg/m^2^, fasting glucose > 125 mg/dL, SBP ≥ 160 mmHg and diastolic blood pressure (DBP) > 100 mmHg or taking antihypertensive medications, hyperlipemia or antilipemic medication; smoking, pregnancy or intending to become pregnant, use of medications, antioxidants, vitamin supplements or adherence to a vegetarian diet, chronic alcoholism, physical activity > 5 h/week, intestinal disorders, anemia (hemoglobin ≤ 13 mg/dL in men and ≤ 12 mg/dL in women), consumption of a research product in the 30 days prior to inclusion in the study, or failure to follow the study guidelines. Participants signed informed consent prior to their participation in the study, which was approved by the Clinical Research Ethical Committee of HUSJ (14-12-18/12aclaassN1), Reus, Spain. The protocol and trial were conducted in accordance with the Helsinki Declaration and Good Clinical Practice Guidelines of the International Conference of Harmonization (GCP ICH) and were reported as CONSORT criteria. The trial was registered at Clinical-Trials.gov: NCT02479568.

### Intervention products

Intervention beverages (supplied by the Florida Department of Citrus, USA) were control drink (CD), an OJ containing 690 mg/L of hesperidin (the natural hesperidin content), and an enriched orange juice (EOJ) containing 1200 mg/L of hesperidin. Ferrer HealthTech (Murcia, Spain) provided the Micronized 2S Hesperidin used in EOJ enrichment. The 2S form, the one present naturally in the OJ, is the most bioavailable [18]. Beverages were analyzed for hesperidin and narirutin content using chromatography–mass spectrometry (LC–MS/MS) (Supporting Information Table S1). Daily doses of 500 mL of CD, OJ and EOJ, provided 0 mg/day, 345 mg/day, and 600 mg/day of hesperidin, and 0 mg/day, 64 mg/day, and 77.5 mg/day of narirutin, respectively. Intervention drinks were similar in appearance and smell, and were differentiated only by a code assigned by an independent researcher not related to the study to guarantee blinding. Flavanone contents of the OJ and the EOJ were stable throughout the study.

### Study design

A randomized, parallel, double-blind, placebo-controlled clinical trial was performed (Supporting Information Fig. S1). Participants were randomly assigned to one of the three intervention groups—CD, OJ, or EOJ—to consume 500 mL/day of the corresponding beverage for 12 weeks. Nested within the sustained consumption study were two dose–response studies, one at baseline and the other after 12 weeks of sustained consumption, where the 500 mL/dose was administered all at once and changes in the outcomes were recorded in the postprandial state. Participants were randomly allocated to the three intervention groups by a computerized random-number generator made by an independent statistician. PROC PLAN (SAS 9.2, Cary, NC: 83 SAS Institute Inc.) with a 1:1:1 allocation using random block sizes of 2, 4, and 6 was used. Participants, researchers and the statistician remained blinded to the type of product administered throughout the study.

After enrolment and following a 1-week run-in period with a control diet consisting of a maintained lifestyle and normal dietary habits based on nutritionist recommendations, the participants started the intervention trial. However, during the intervention period, the participants were instructed to also maintain their dietary habits, to completely refrain from consuming citrus-containing foods and to limit their total intake of flavonoid-rich foods (tea, coffee, cocoa, wine and other fruit juices) to reduce the possible masking effects that can exert these foods on BP [22, 23]. During the sustained study, participants attended seven visits (V) at the HUSJ-Eurecat. Dose–response postprandial studies, performed at V1 and V7, lasted from 08:00 a.m. to 02:00 p.m., and participants received a light meal before leaving. In addition to the baseline (0 h), blood samples were collected at 2 h, 4 h, and 6 h after the single dose of 500 mL. The adherence of the volunteers to their dietary habits throughout the study was assessed by a 3-day food record at V1, V3, V5, and V7. At each visit, subjects underwent a physical examination by a general practitioner and completed a Physical Activity Questionnaire Class AF [24], and anthropometric measurements were recorded. Samples were stored at -80ºC in the central laboratory’s Biobanc of HUSJ-Eurecat (biobanc.reus@iispv.cat) until required for batch analyses.

### Compliance measures

The plasma levels of the following biomarkers of nutrient exposures were measured by LC–MS/MS in the plasma samples: hesperetin-7-*O*-β-d-glucuronide, hesperetin-3-*O*-β-d-glucuronide, hesperetin-7-*O*-sulfate, naringin-4-*O*-β-d-glucuronide, naringin-glucuronide and naringin sulfate. The extraction was carried out with a semi-automated process using Agilent Bravo Automated Liquid Handling Platform. Briefly, 20 μL of internal standard (Hesperetin d4) was mixed with 125 μL of plasma and 750 μL of methanol. The mixture was vortexed and centrifuged at 4700 rpm at 4 °C, and then 900 μL was evaporated in a Speed-Vac at room temperature. Residues were reconstituted in 25 μL of MeOH and 75 μL of H_2_O (1% of HFor) and injected in the LC–MS/MS, an Agilent 1200 series ultra-high-performance liquid chromatography (UHPLC) system coupled to a 6490 Triple Quad mass spectrometer, with electrospray source ionization (ESI) operating in negative mode.

### Main outcome measures

SBP and DBP were measured twice after 2–5 min of respite, with the patient in a seated position, with 1-min interval between, using an automatic sphygmomanometer (OMRON HEM-907; Peroxfarma, Barcelona, Spain). The mean values were used for statistical analyses. Office PP, which represents the force that the heart generates each time it contracts, was determined by the difference between SBP and DBP [25]. The main outcomes were measured in both dose–response and sustained consumption studies.

### Secondary outcomes

Homocysteine in serum samples was determined by LC–MS/MS. F2α isoprostanes were determined by a quantitative sandwich enzyme-linked immunosorbent assay (ELISA) (Caymanchem, MI, USA) in 24-h urine. Soluble Intercellular Adhesion Molecule-1 (ICAM-1) and Soluble Vascular Cell Adhesion Molecule-1 (VCAM-1) were determined in serum by the Luminex™xMAP technology with the EPX010-40,232-901 kit eBioscience (Thermo Fisher Scientific, Waltham, Massachusetts, USA), in the the Bio-Plex™ 200 instrument (Bio-Rad, Hercules, California, USA). Uric acid was measured by standardized methods on an autoanalyzer 182 (Beckman Coulter-Synchron, Galway, Ireland) in serum samples. All biological biomarkers were measured in the sustained consumption study. Homocysteine was additionally measured after the single 500-mL dose of the corresponding intervention product in both dose–response studies.

### Transcriptomic analyses

Gene expression was assessed in peripheral blood mononuclear cells (PBMCs) with an Agilent Microarray Platform (Agilent Technologies, Santa Clara, California, USA) in a subsample (*n* = 37) of participants (11, 15, and 11, in CD, OJ, and EOJ groups, respectively) at baseline and after 12 weeks. PBMC RNA was isolated using Ficoll gradient separation GE Healthcare Bio Sciences, Barcelona, Spain), RNA yield was quantified with a Nanodrop UV–VIS Spectrophotometer and integrity was measured with an Agilent 2100 Bioanalyzer using the Total RNA Nano kit and the Eukaryote Total RNA Nano (Agilent Technologies, Santa Clara, California, USA). Total RNA from the PBMCs was labeled with one color (Cy3) (ref: 5190-2305, Agilent Technologies, Santa Clara, California, USA) and hybridized using a Gene Expression Hybridization Kit (ref: 5188-5242, Agilent Technologies, Santa Clara, California, USA). Image scanning was performed with an Agilent Microarray Scanner System with SureScan High Resolution Technology (Agilent Technologies, Santa Clara, California, USA). Differentially expressed genes were subjected to functional and biochemical pathway analysis using Gene Ontology, Kyoto Encyclopedia of Genes and Genomes (KEGG) (https://www.genome.jp/kegg) and PANTHER (protein annotation through evolutionary relationship classification system (https://www.pantherdb.org/) [26] biochemical pathway databases. The analysis was performed using GeneCodis (https://www.genecodis.dacya.ucm.es [27] software.

Selected genes related to hypertension were validated by PCR. Briefly, to analyze the expression of the genes and validate the DNA array results, cDNA was synthesized using the High-Capacity cDNA Reverse Transcription Kit (Applied Biosystems, 4 Barcelona, Spain) and MyGene Series Peltier Thermal Cycler (LongGene Scientific, Zhejiang, China) and used for reverse transcription. The cDNA was subjected to quantitative reverse transcription-polymerase chain reaction amplification using LightCycler 480 SYBR Green I Master (Roche Diagnostic, Sant Cugat del Vallès, Barcelona, Spain) and a LightCycler 480 II system (Roche Diagnostic, Sant Cugat del Vallès, Barcelona, Spain).

### Sample size and power analyses

A sample size of 159 individuals was calculated assuming an expected dropout rate of 20% and a type I error of 0.005 (two sided), which allows at least 80% power for the detection of statistically significant differences in the SBP of 4 mmHg among the groups. The population standard deviation of the SBP was estimated to equal 6 mmHg [28].

### Statistical analyses

Descriptive data were expressed as the mean 95% confidence interval (CI). The normality of variables was assessed by the Kolmogorov–Smirnov test. Non-parametric variables were log transformed. ANOVA was used to determine differences in baseline characteristics. Analyses were made by intention-to-treat. Multiple imputation was made by linear regression analysis. Intra-treatment comparisons were performed by means of a general linear model with Bonferroni correction and age and sex as covariables. Inter-treatment comparisons were carried out by analysis of covariance (ANCOVA) model adjusted for age and sex. For transcriptomic analyses, quality control was performed through principal component analyses. Statistical comparisons were performed by Student’s *t* test or Welch’s *t* test if proceeded. Multiple testing correction was performed using the Benjamini–Hochberg False Discovery Rate (FDR) control procedure. Probes were assumed to be differentially expressed if they presented a *P* value < 0.05 and a fold change ≤ −0.58 or ≥ 0.58 in log2 scale (corresponding to 1.5-fold difference in natural scale). Calculations were performed using the R statistical language. Comparisons among treatments were carried out by an ANCOVA model adjusted by age and sex and baseline values. Statistical significance was defined as a *P* value ≤ 0.05 for a two-sided test. Analyses were performed using SPSS for Windows, version 21 (IBM corp., Armonk, NY, USA). All data were analyzed according to the pre-specified protocol.

## Results

### Study participants

**Fig. 1:**
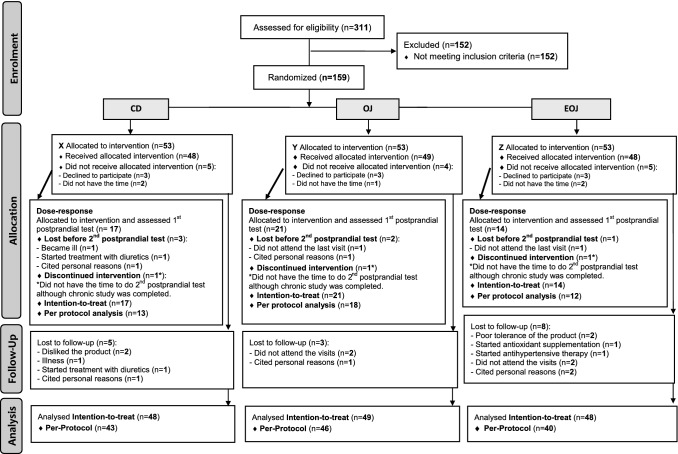
Flow chart of the study

Of the 311 subjects who were assessed for eligibility, 152 did not meet the inclusion criteria and were excluded. The remaining 159 participants were randomly allocated to the CD, OJ, and EOJ groups, (*n* = 53 in each group). Ultimately, 129 participants completed the study (43 in the CD, 46 in the OJ, and 40 in the EOJ groups) (Fig. 1). For the dose–response study, of the 52 allocated participants, three discontinued the intervention from the beginning, and six were lost for the second dose–response study. Thus, 52 participants (17 in the CD, 21 in the OJ, and 14 in the EOJ groups) were available for the first dose–response study, and 43 (13 in the CD, 18 in the OJ, and 12 in the EOJ groups) were available for the second dose–response study (Fig. 1). No differences in baseline characteristics were observed among the groups (Supporting Information Table S2). The baseline characteristics of participants in the dose–response study were similar to those of the whole sample. No changes in the level of physical activity were observed from the beginning to the end of the study in any group (data not shown). No differences in dietary intake were observed among groups with exception of protein (% energy) intake, which was greater in the OJ group than in the EOJ one (*P* = 0.031) (Supporting Information Table S3).

### Compliance biomarkers

The volunteer compliance intervention was considered optimal because all the compliance biomarkers (hesperetin-7-*O*-β-d-glucuronide, hesperetin-3-*O*-β-d-glucuronide, hesperetin-7-*O*-sulfate, naringerine-4-*O*-β-d-glucuronide, naringenin-glucuronide and naringenin-sulfate) increased significantly during the OJ and EOJ intervention compared with the baseline values and the CD group intervention. At 12 weeks, the metabolite hesperetin-7-β-d-glucuronide was the main differentially expressed metabolite between the OJ and EOJ groups and the CD group (*P* < 0.001).

**Fig. 2:**
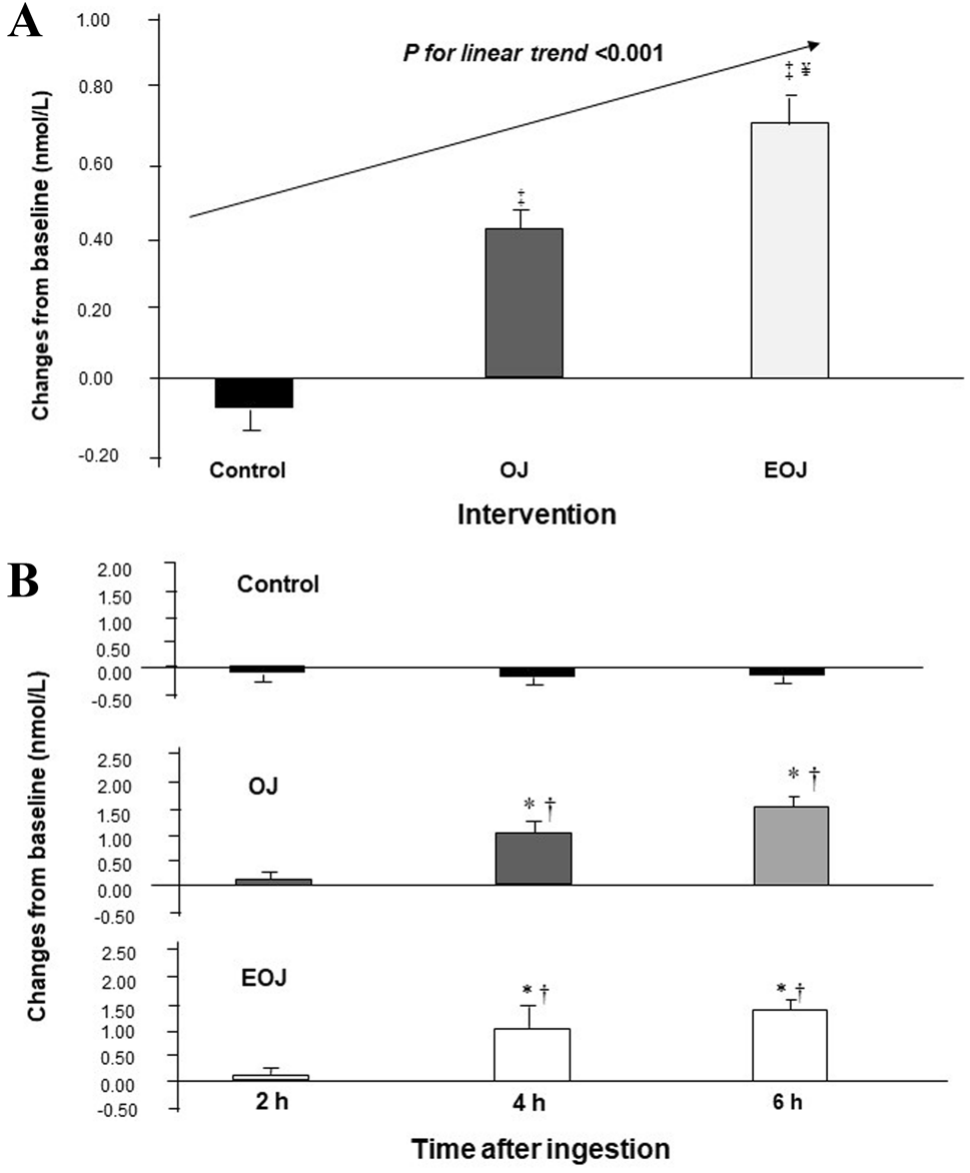
Changes in plasma hesperitin-7-β-d-glucuronide after ingestion of control, orange juice (OJ), and enriched OJ. **a** After sustained consumption for 12 weeks (500 mL/day). **b** At the beginning of the study after a single dose of 500 mL. *P < 0.05 versus baseline; ^†^*P* < 0.05 versus control group; ^‡^*P* < 0.001 versus control; ^¥^*P* < 0.05 versus OJ

After 12 weeks of treatments (Fig. 2a), plasma hesperetin-7-β-d-glucuronide increased in a dose-dependent manner with the hesperidin content of the beverage administered (*P* < 0.001 for linear trend), and the increase in the EOJ group was significantly higher than that of the OJ group (*P* < 0.05). In the dose–response studies, plasma hesperetin-7-β-d-glucuronide increased at 4 and 6 h after OJ and EOJ (*P* < 0.005 versus changes in CD), both at the beginning (Fig. 2b) and at the end of the study. The individual changes in plasma hesperetin-7-β-d-glucuronide are depicted in Supplementary Fig. 4 .

### Main outcomes

**Fig. 3:**
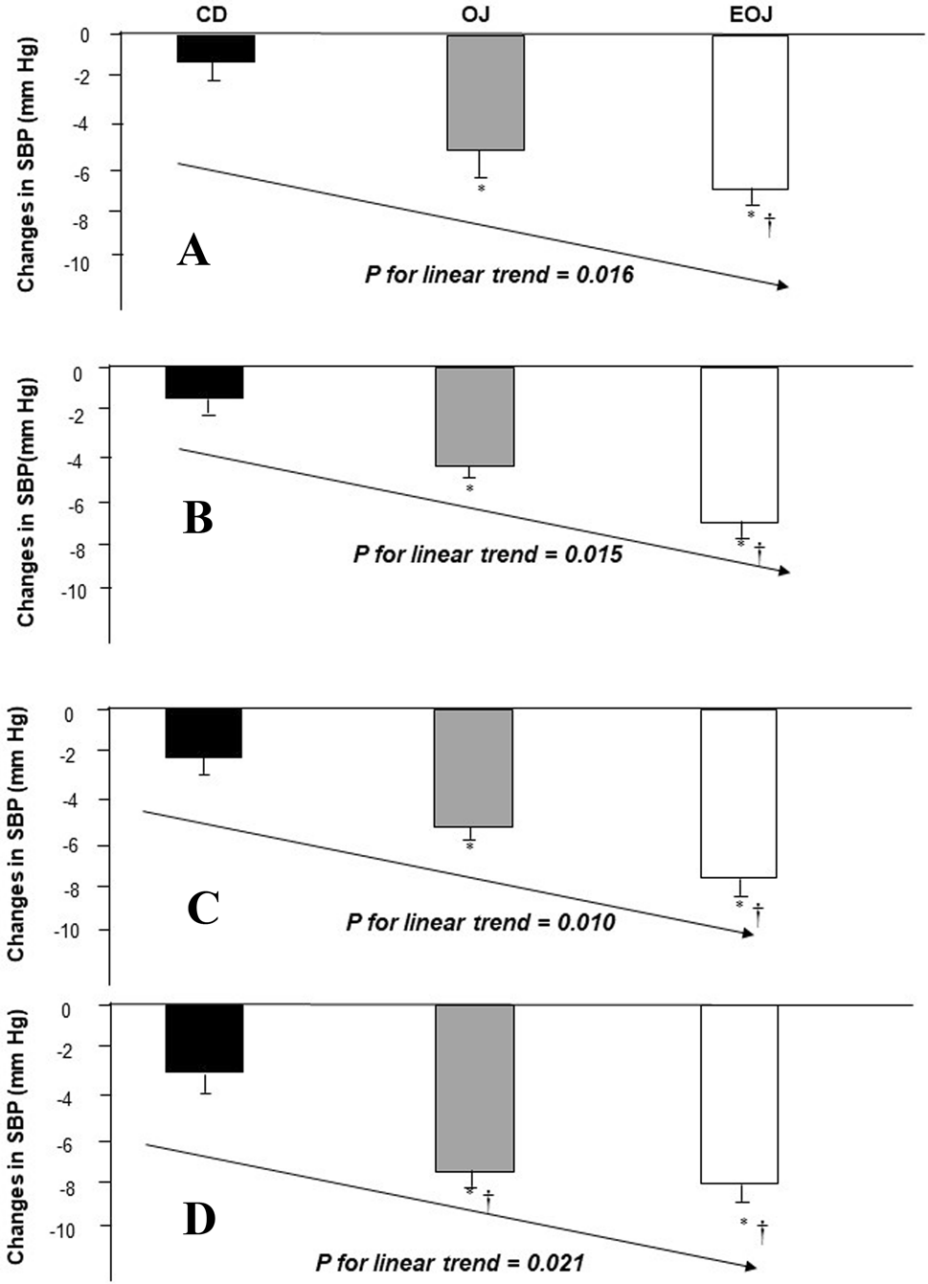
Changes in systolic blood pressure (SBP) at 2 (**a**), 6 (**b**), 10 (**c**), and 12 (**d**) weeks after sustained consumption of control drink (CD), orange juice (OJ), and hesperidin-enriched OJ (EOJ). *P < 0.05 versus baseline; ^†^*P* < 0.05 versus CD

**Fig. 4:**
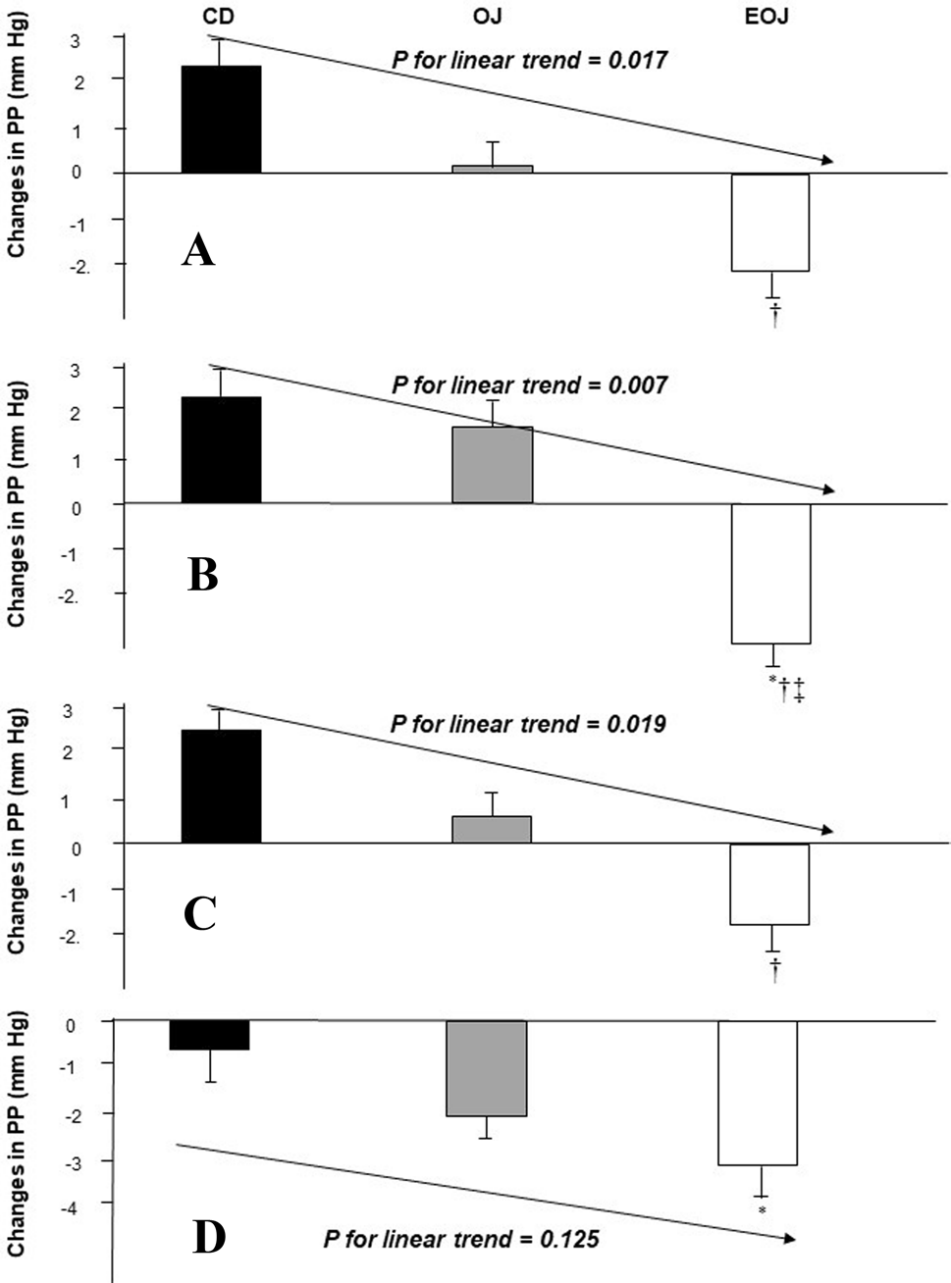
Changes in pulse pressure (PP) at 2 (**a**), 6 (**b**), 10 (**c**), and 12 (**d**) weeks after sustained consumption of control drink (CD), orange juice (OJ), and hesperidin-enriched OJ (EOJ). ^*^P < 0.05 versus baseline; ^†^*P* < 0.05 versus CD; ^‡^P < 0.05 versus OJ

**Fig. 5:**
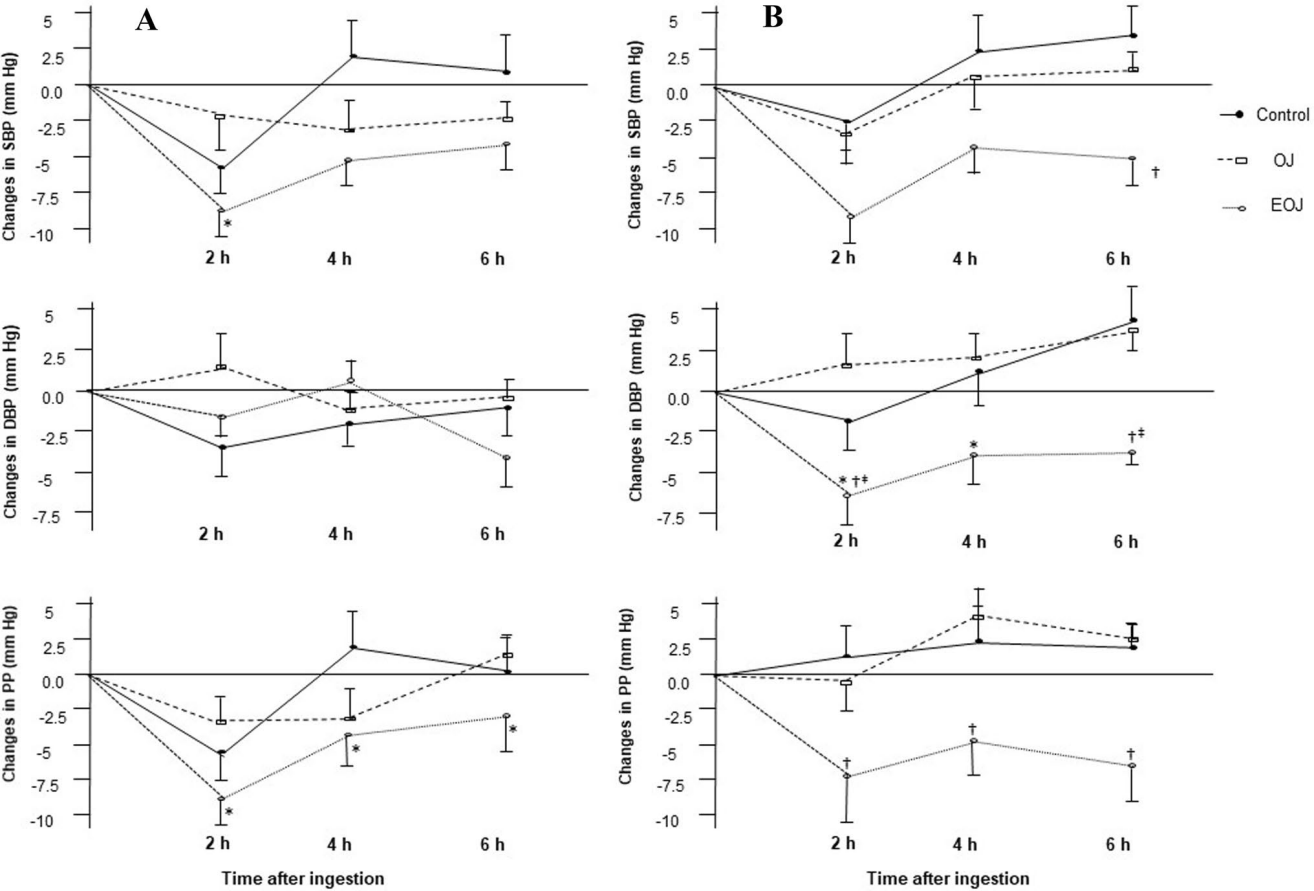
Changes in systolic blood pressure (SBP), diastolic blood pressure (DBP), and pulse pressure (PP) after a single dose of 500 mL of control drink, orange juice (OJ), and enriched OJ at the beginning (**a**) and at the end of the study (**b**)^a^

Changes in SBP at 2, 6, 10 and 12 weeks are shown in Fig. 3. SBP decreased in a dose-dependent manner with the hesperidin content of the beverage administered (*P* < 0.05 for linear trend). SBP decreased at weeks 4, 8, and 12 after OJ consumption, the decreases reaching significance versus changes.

in the CD at week 4 and 12 (*P* < 0.05) by mean 95% IC −5.58 (−9.8; −1.3) mmHg and −5.06 (−8.8; −1.3) mmHg, respectively. A borderline significance at week 8 was also observed (*P* = 0.056). After consumption of EOJ, SBP decreased in all evaluated weeks compared to CD, the decreases reaching significance (*P* < 0.05) at all weeks, with the only exception of week 8 in which a borderline significance (*P* = 0.078) was observed. The average of all decreases through the study was −6.35 and −7.36 mmHg for OJ and EOJ interventions, respectively. DBP decreased similarly after all interventions and in all weeks (*P* < 0.05) (data not shown). Changes in PP through the study are shown in Fig. 4. PP decreased in a dose-dependent manner with the hesperidin content of the beverage administered (*P* < 0.05 for linear trend) in all weeks, but in the 12 week, the trend did not reach significance (*P* = 0.125). Concerning dose–response studies, at the beginning of the study (Fig. 5a), significant decreases were observed in SBP at 2 h and in PP at all evaluated times (*P* < 0.05) after a single dose of 500 mL only in the case of EOJ. No changes were observed in DBP values. After 12 weeks of treatment (Fig. 5b), a single dose of 500 mL resulted in changes in BP and PP also only in the EOJ group (Panel B). DBP decreased versus baseline at all evaluated times (*P* < 0.05), and the decrease at 2 h and 6 h reached significance versus changes in CD (*P* < 0.05). Additionally, the observed decreases in SBP at 6 h and those of PP at all evaluated times reached significance versus changes in CD (*P* < 0.05). At 4 h and 6 h postprandial after our, an inverse relationship was observed between hesperidin-7-β-d-glucuronide values and those of PP (*R* = −0.354, *P* = 0.023, and *R* = −0.377, *P* = 0.015, respectively). At 6 h, an inverse relationship was also observed between the increase in hesperidin-7-β-d-glucuronide and SBP values (*R* = −0.353, *P* = 0.024).

### Secondary outcomes

At week 12 after sustained consumption, homocysteine values decreased after OJ and EOJ treatments, and the decreases reached significance versus changes after the CD treatment. At this time point, homocysteine plasma values decreased in the postprandial state at 2 h and 4 h after OJ and after 2 h of EOJ ingestion (*P* < 0.05) (Fig. S2 in the online-only Data Supplement). Uric acid decreased at 12 weeks after EOJ treatment, reaching significance versus changes in the CD treatment (*P* = 0.044). ICAM-1 decreased at week 12 after EOJ treatment (*P* = 0.032), but no changes were observed between treatments. At week 12 after sustained consumption, uric acid concentrations were directly related to SBP, DBP, and PP (*P* < 0.05). No changes were observed in other secondary outcomes. After 12 weeks sustained consumption, the values of SBP directly correlated with those of ICAM-1 (*R* = 0.251, *P* = 0.004) and VCAM (*R* = 0.185, *P* = 0.036) (Fig. S3 in the online-only Data Supplement), and the decrease in F2-isoprostanes, although without significance, were directly correlated with the decreases in SBP (*R* = 0.178, *P* = 0.042).

No adverse events were reported. All products were well tolerated.

**Fig. 6:**
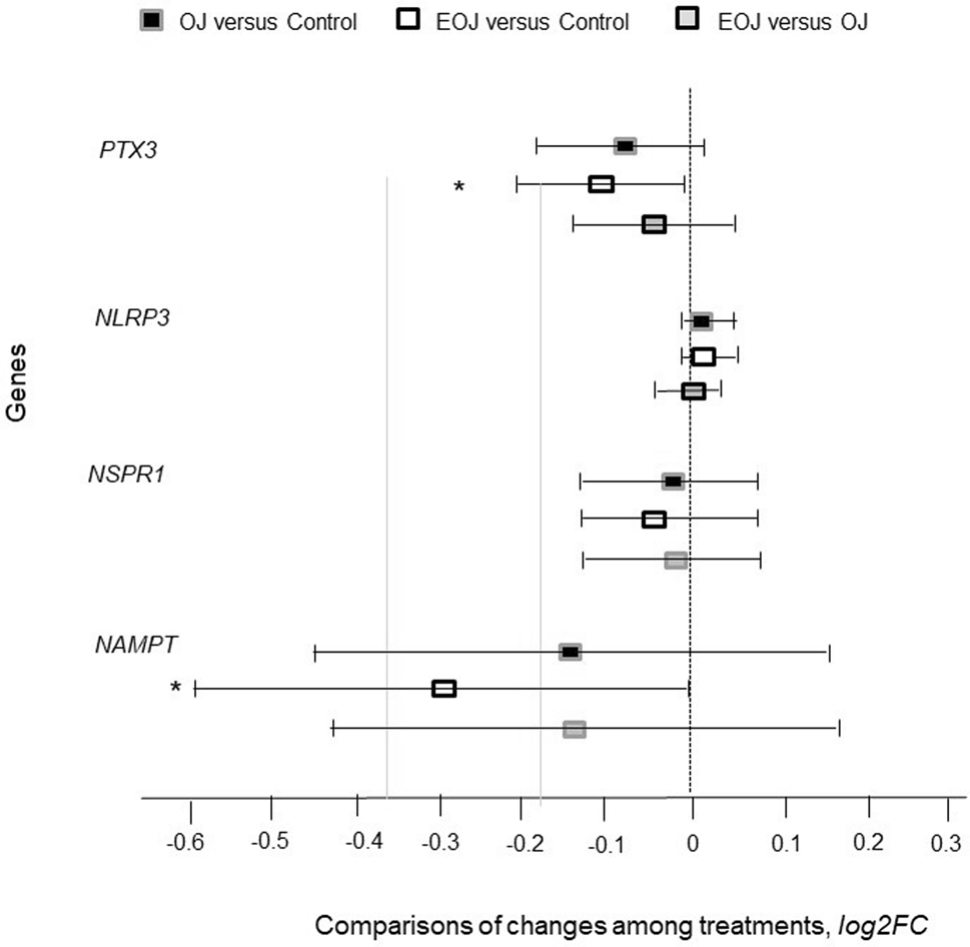
Comparisons of changes in gene expression (*log2FC*, mean (95%CI) among treatments after interventions. *PTX3,* Pentraxin-3; *NLRP3,* NLR family, pyrin domain containing 3; NPSR1, neuropeptide S receptor 1; and *NAMPT,* nicotinamide phosphoribosyl transferase. The dot axis displays the significance between orange juices at the *P* < 0.05 level. *P < 0.05

In transcriptomic analyses, after the sustained consumption study, four genes related to hypertension were identified: Pentraxin-3 (*PTX3*); NLR family, pyrin domain containing 3 (*NLRP3*); neuropeptide S receptor 1(NPSR1); and nicotinamide phosphoribosyl transferase (*NAMPT*), which were differentially expressed after 12 weeks of treatment. The expressions of the *PTX3* and *NAMPT* genes decreased significantly in PBMC after the EOJ intervention versus the control treatment (*P* < 0.05). Figure 6 shows the comparisons among interventions considering the dot axis at *P* < 0.05 to be of significance. The decreases in SBP and PP at week 12 were directly related to the decrease in *PTX3* expression (*R* = 0.393, *P* = 0.016 and *R* = 0.487, *P* = 0.002, respectively). The decrease in PP at week 12 was directly related to that of *NAMPT* expression (*R* = 0.344, *P* = 0.037). Although no significance was observed, changes in *NRLP3* expression were inversely related to those of PP at week 12 (*R* = −0.420, *P* = 0.010). In turn, expression of *PTX3* at week 12 was directly related to that of *NAMPT* (*R* = 0.759, *P* < 0.001).

## Discussion

In the present study, on the one hand, SBP and PP decreased in a dose-dependent manner with the hesperidin content of the beverage administered throughout the 12 weeks of the study. On the other hand, a single dose of 500-mL EOJ, but no other treatment, reduced SBP, and PP, greater changes when the dose was administered at the end of the study after 12 weeks of sustained treatment where DBP changes were also observed. Thus, these suggested that sustained consumption of hesperidin optimizes acute BP-lowering effects.

After 12 weeks, sustained EOJ consumption-related decreases in uric acid and ICAM were observed. Homocysteine decreased at 12 weeks after OJ and EOJ, and postprandial decreases in homocysteine were also present after single doses of OJ and EOJ at the end of the study. In agreement with the decrease in SBP and PP, *PTX3* and *NAMPT* gene expression decreased in PBMCs at 12 weeks after sustained EOJ treatment.

Currently, the worldwide prevalence of hypertension exceeds 1.3 billion [29] and is the main risk factor for death and disability-adjusted life-years lost during 2010 [30]. A 10-mmHg SBP decrease is associated with reductions of 22 and 41% in coronary heart disease and stroke, respectively [31]. Decreases in SBP with medical therapies range from 5 to 15 mmHg [32]. The average reductions in SBP throughout our study were −6.35 and −7.36 mmHg for the OJ and EOJ interventions, respectively. Our data are in agreement with those obtained after 8–12 weeks of a treadmill exercise program in hypertensive individuals (6.2 mmHg) [33] and after consumption of the Dietary Approaches to Stop Hypertension (DASH) diet (6.74 mmHg) [34] as well as with the results of a meta-analysis reporting a mean reduction of 8 mmHg SBP by regular endurance exercise in hypertensive patients [35]. PP, a surrogate marker of aortic stiffness, is recognized as a powerful and independent risk factor for CVD with prognostic utility beyond BP measurements [36, 37]. Throughout our study, the average PP reductions was −2.41 mmHg after EOJ. A 10-mmHg increase in PP is associated with a 13% increase in all-cause mortality and >20% increase in recurrent myocardial infarction [36].

Throughout the 12 weeks of sustained intervention with 345 mg/day of hesperidin in OJ and 600 mg/day of hesperidin EOJ, we observed decreases in SBP and PP, but not in DBP. Our results are opposite of those reported in overweight men, with a DBP-lowering effect but not an SBP one, after 4 weeks of OJ or a hesperidin-rich capsule providing 292 mg and 146 mg of hesperidin/day, respectively [23]. No benefits on BP were reported after sustained high hesperidin consumption of 549 mg/L/day over 8 weeks in healthy elderly individuals [17]; 6 weeks at 420 mg/day in healthy volunteers [18]; or 3 weeks at 500 mg/day in individuals with metabolic syndrome [38]. In type 2 diabetes patients, however, consumption (500 mg/day) of hesperidin over 6 weeks led to decreases in SBP and DBP [16]. Differences in populations and lengths of treatment could account for discrepancies among studies. If the objective of a study is to improve a specific cardiovascular risk factor, subjects that present symptoms associated with the specific cardiovascular risk factor should be included in the study [39]. Thus, the present study constitutes the first RCT that assesses the effects of hesperidin on BP and PP in pre- and stage-1 hypertensive subjects; whereas, the populations studied in other RCTs were overweight, obese or diabetic with no hypertension or elevated BP levels [40]. At present, few reports exist concerning the effect of hesperidin on PP. Hesperidin reversed aortic stiffness in mice [41]. Although the beneficial effect of flavonoids on arterial stiffness is emerging [42], our data are the first available to supporting the effect of dietary flavanones on human arterial stiffness.

Mechanisms by which hesperidin could contribute to the control of BP and PP are associated with improvements on endothelial function, oxidative stress, and inflammation [8]. Homocysteine is associated with these risk factors and with a renin–angiotensin system activation to induce a BP increase [43]. In agreement with this, we observe a decrease in homocysteine concomitant with decreases in BP and PP after hesperidin treatments. After 12-week EOJ consumption, ICAM-1 values decreased, and this decrease and those of other inflammatory and oxidative markers were directly related to the SBP decrease. In our work, plasma uric acid decreased after 12-week EOJ consumption. Hyperuricemia is strongly associated with hypertension and arterial stiffness through activation of the NLPR3 inflammasome [44]. Accordingly, in our study changes in *NLPR3* gene expression after 12 weeks were inversely associated with those of PP. After 12 weeks of EOJ consumption, we observed a decrease in PBMC expression of two key hypertension-related genes: *PTX3* and *NAMPT*. Serum levels of PTX3, a marker of inflammation activation, are elevated in hypertensive patients [45], and experimental studies reported a direct role of PTX3 in vascular function and BP homeostasis [46]. NAMPT, also called visfatin, is secreted by visceral fat and is a stimulator of proinflammatory cytokines [47]. NAMPT is elevated not only in hypertensive patients but also in pre-hypertensive patients [48, 49], leading to the proposal that NAMPT is a marker for damage in the pre-hypertensive state [48]. Thus, in our study, the decrease in biochemical and transcriptomic markers could account for the decreases in BP and PP after intake of hesperidin-rich beverages.

One factor that could minimize differences among sustained hesperidin interventions could be the similar contribution of the narirutin present in these treatments. In experimental and human studies, naringin and narirutin showed a hypotensive effect [12, 50, 51]. When comparing the dose–response results on the main outcomes, however, only the EOJ single-dose intervention was capable of decreasing SBP, DBP, and PP at the postprandial level. At present, few reports exist concerning the dose–response effect of hesperidin consumption in humans. No changes in SBP or DBP have been reported at 5 h after OJ or hesperidin supplementation (containing 320 mg of hesperidin) in healthy elderly individuals, despite an increase in hesperidin at this time point [19]. The fact that a unique measurement was obtained after a single dose could explain differences between studies. To the best of our knowledge, our data are the first to report the postprandial benefits of a hesperidin-enriched beverage to BP and PP, as well as the fact that its sustained consumption enhances these benefits.

The study has strengths and limitations. As a strength, the participants’ diets were monitored throughout the entire study, and avoiding hesperidin intake and limiting the consumption of flavonoid-rich foods were given as dietary recommendations to all the participants, which is of special interest in nutritional RCTs because these guidelines would limit confounding between other dietary compounds and the dietary intervention [40]. The dietary recommendations were established equally for all the intervention groups (CD, OJ and EOJ), and thus, the possible changes in the metabolome profile and consequently the downstream effects on BP due to these dietary modifications would be equally observed in all the groups, which would result in the control of these changes. Another important strength is that this study constitutes the first human RCT that assessed a compliance marker, hesperitin-7-β-d-glucuronide metabolite, which is associated with PP and SBP values, and thus, these results add robustness to our study.

Multiple measurements throughout the study permitted the assessment of the homogeneity of the results. One limitation is the inability to assess potential interactions between the interventions and other dietary components. Additionally, a larger sample size could have permitted detection of significant differences between both hesperidin treatments. Although BP measurements were performed with maximal care, a 24-h ambulatory BP monitoring could have been more accurate. The fact that participants were pre- and stage-1 hypertensive individuals limits the extrapolation of the results to the general population. Whether additional or different effects would have been observed over longer time periods is unknown, but longer intervention periods could have affected the compliance of the individuals.

In summary, our results show that the intake of hesperidin in OJ decreases SBP and PP after sustained consumption in a dose-dependent manner with the hesperidin content of the beverage administered. Chronic consumption of hesperidin-rich OJ enhances the postprandial response of decreasing SBP, DBP and PP. Decreases in homocysteine, uric acid and inflammatory markers at the systemic level and in *PTX3* and *NAMPT* at the transcriptomic level could account for the observed changes in BP and PP.

### Perspectives

In a randomized, controlled clinical trial with pre- and stage-1 hypertensive individuals, we showed that sustained consumption of hesperidin promoted a dose-dependent decrease in SBP and PP with the hesperidin content of the beverage administered. Our data are the first to support an effect of dietary flavanones on human arterial stiffness. Additionally, we report for the first time the postprandial benefits of a hesperidin-enriched beverage to BP and PP, as well as the fact that its sustained consumption enhances these benefits. Regular consumption of OJ, particularly hesperidin-rich OJ, could be a useful co-adjuvant tool for BP management in pre- and stage-1 hypertensive individuals. This fact has public health implications in preventive medicine for reducing the secondary effects of long-term medical treatment of mild hypertension.

## Electronic supplementary material

Below is the link to the electronic supplementary material.

Supplementary file1 (DOCX 32 kb)

Supplementary file2 (PPT 159 kb)

Supplementary file3 (PPT 585 kb)
