# Supplementary material for: Effects of hesperidin in orange juice on blood and pulse pressures in mildly hypertensive individuals: a randomized controlled trial (Citrus study)
Source: Eur J Nutr. 2020 Jul 13;60(3):1277–88. doi: 10.1007/s00394-020-02279-0 (PMC7987641; doi:10.1007/s00394-020-02279-0)
Supplement: Supplementary file 1 — Supplementary file1 (DOCX 32 kb) [file 394_2020_2279_MOESM1_ESM.docx]

**Online Supplemental Tables for the manuscript**

**Effects of hesperidin in orange juice on blood and pulse pressures in mildly hypertensive individuals: a randomized controlled trial**

Rosa M. Valls^1,2^, Anna Pedret^2,1^*, Lorena Calderón-Pérez^1,2^, Elisabet Llauradó^1,2^, Laura Pla-Pagà^2,1^, Judit Companys^2,1^, Ana Moragas^3,4,5^, Francisco Martín-Luján^3,4,6^, Yolanda Ortega^4,5,7^, Montse Giralt^1^, Marta Romeu^1^, Laura Rubió^1^, Jordi Mayneris-Perxachs^2,8^, Núria Canela^8^, Francesc Puiggrós^2^, Antoni Caimari^2^, Josep M. Del Bas^2^, Lluís Arola^2,9^, Rosa Solà^1,2,10^.

*^1^Universitat Rovira i Virgili, Facultat de Medicina i Ciències de la Salut,* *Functional Nutrition, Oxidation, and Cardiovascular Diseases Group (NFOC-Salut), Reus, Spain.*

*^2^Eurecat, Centre Tecnològic de Catalunya, Unitat de Nutrició i Salut, Reus, Spain.*

*^3^Universitat Rovira i Virgili, Departament de Medicina i Cirurgia, Reus, Spain*

*^4^Institut Universitari d’Investigació en Atenció Primària-IDIAP Jordi Gol, Tarragona, Spain.*

*^5^Primary Care Centre Jaume I, Institut Català de la Salut, Tarragona, Spain.*

*^6^Primary Care Centre El Morell, Institut Català de la Salut, Tarragona, Spain.*

*^7^Primary Care Centre Salou, Institut Català de la Salut, Tarragona, Spain.*

*^8^Eurecat, Centre Tecnològic de Catalunya, Centre for Omic Sciences, Reus, Spain*

*^9^Universitat Rovira i Virgili, Departament de Bioquímica i Biotecnologia, Grup de Recerca en Nutrigenòmica, Tarragona, Spain.*

*^10^Hospital Universitari Sant Joan de Reus, Reus, Spain.*

***Corresponding Author:** Anna Pedret, PhD. Eurecat, Centre Tecnològic de Catalunya. Unitat de Nutrició i Salut. Av. de la Universitat, 1, 43204 Reus, Spain**.** Tel: (+34) 977 75 14 84**.** E-mail: [anna.pedret@eurecat.org](mailto:anna.pedret@eurecat.org)

**Table S1. Composition of the intervention products calculated by 500 mL/day***

|  | Intervention | | |
| --- | --- | --- | --- |
|  | CD | OJ | EOJ |
| Acidity, % | 2.49 | 2.49 | 2.49 |
| Sugar, g | 43.1 | 37.7 | 37.7 |
| Vitamin C, mg | 235.3 | 235.3 | 235.3 |
| Citric acid, g | 3.40 | 3.40 | 3.40 |
| Narirutin, mg | ND | 64 | 77 |
| Hesperidin, mg | ND | 392 | 670 |

*In frozen concentrated canned drink, once diluted 3.4:1 (water to syrup). Abbreviations: CD, control drink; OJ, orange juice; EOJ, enriched orange juice; kcal, kilocalories; ND, non-detectable.

**Table S2. Baseline characteristics of participants by intervention group**

| Variable | CD  (n = 53) | OJ  (n= 53) | EOJ  (n= 53) | P |
| --- | --- | --- | --- | --- |
| Age, *y* | 45.4 ±13.0 | 43.3 ± 12.0 | 43.6 ± 11.8 | 0.629 |
| Females, *%* | 34.0 | 32.1 | 34.0 | 0.981 |
| SBP, *mm Hg* | 132 ± 9.94 | 132 ± 9.11 | 134 ± 9.82 | 0.687 |
| DPB, *mm Hg* | 79 ± 8.14 | 80 ± 8.42 | 79 ± 10.2 | 0.868 |
| Pulse pressure, *mm Hg* | 53 ± 9.09 | 52 ± 8.05 | 54 ± 6.74 | 0.261 |
| Weight, *kg* | 77.3 ± 15.4 | 78.8 ± 12.2 | 75.9 ± 11.6 | 0.523 |
| BMI, *kg/m^2^* | 26.1 ± 3.8 | 26.4 ± 3.6 | 26.1 ± 3.3 | 0.858 |
| Waist circumference, *cm* | 93.0 ± 11.0 | 91.7 ± 10.9 | 91.4 ± 10.7 | 0.766 |
| Waist/height, *cm* | 0.54 ± 0.06 | 0.53 ± 0.07 | 0.54 ± 0.07 | 0.790 |
| Conicity index | 1.50 ± 0.76 | 1.30 ± 0.35 | 1.39 ± 0.62 | 0.269 |
| Glucose, *mg/dL* | 91.6 ± 9.2 | 93.6 ± 11.6 | 93.6 ± 9.6 | 0.517 |
| Cholesterol*, mg/dL* |  |  |  |  |
| Total | 196 ± 30.1 | 198 ± 32.7 | 196 ± 31.6 | 0.937 |
| LDL | 124 ± 26.4 | 125 ± 31.5 | 127 ± 25.1 | 0.900 |
| HDL | 50.9 ±13.4 | 51.0 ± 14.7 | 49.8 ± 13.0 | 0.889 |
| Triglycerides^*^, *mg/dL* | 82 (67-118) | 85 (65-121) | 81 (63-116) | 0.624 |
| Physical activity, *AU* | 3.08 ± 0.06 | 3.12 ± 1.38 | 3.12 ± 1.26 | 0.986 |

Data are expressed as the mean ± standard deviation, or percentages. CD, control drink; OJ, orange juice; EOJ, enriched orange juice; SBP, systolic blood pressure; DBP, diastolic blood pressure; Pulse pressure = SBP-DBP; BMI, body mass index; LDL, low-density lipoproteins; HDL, high-density lipoproteins * median (25^th^ -75^th^ percentiles). AU, arbitrary units: 0, inactive; 1, very low activity; 2, low activity; 3, moderately active; 4, very active. *P* for ANOVA with logarithmic transformation for triglycerides.

| **Table S3. Energy, nutrients, fibre and alcohol after 12 weeks of intervention** | | | | |
| --- | --- | --- | --- | --- |
| **Variable** | **Treatment** | | | **P*** |
|  | **Control P** | **OJ P** | **Enriched OJ P** |  |
| Energy, *kcal/day*  Baseline  12-week | 2443 ± 638  2270 ± 691 0.033 | 2422 ± 618  2154 ± 610 0.001 | 2488 ± 627  2293 ± 565 0.019 | NS |
| HC, *% energy*  Baseline  12-week | 37.5 ± 6.3  34.0 ± 8.4 0.008 | 37.9 ± 5.8  35.8 ± 7.5 0.042 | 37.5 ± 6.1  36.8 ± 7.9 0.533 | NS |
| HC, *grams*  Baseline  12-week | 224 ± 55  187± 66 <0.001 | 227± 67  190 ± 62 <0.001 | 227 ± 66  205 ± 61 0.001 | NS |
| Protein, *% energy*  Baseline  12-week | 17.6 ± 2.9  17.9 ± 3.6 0.578 | 16.6 ± 2.4  17.7 ± 3.5 0.028 | 17.6 ± 3.8  16.8 ± 2.9 0.142 | 0.031**^†^** |
| Protein, *grams*  Baseline  12-week | 104 ± 29.5  100 ± 32.6 0.287 | 99.0 ± 26.0  93.8 ± 28.6 0.204 | 106 ± 28.9  94.3 ± 2.,3 0.003 | NS |
| Total fat, % *energy*  Baseline  12-week | 43.0 ± 5.2  45.5 ± 7.7 0.036 | 43.1 ± 5.7  43.8 ± 6.1 0.496 | 41.9 ± 4.8  43.1 ± 5.2 0.230 | NS |
| Total fat, *grams*  Baseline  12-week | 120 ± 42.4  118 ± 44.8 0.759 | 118 ± 33.8  107 ± 36.7 0.032 | 118 ± 34.7  112 ± 32.6 0,310 | NS |
| SFA, *% energy*  Baseline  12-week | 12.1± 2.4  12.6 ± 3.1 0.233 | 12.4 ± 2.8  13.3 ± 3.3 0.092 | 12.0 ± 2.7  11.9 ± 2.3 0.886 | NS |
| SFA, *grams*  Baseline  12-week | 34.7± 14.8  33.8 ± 15.4 0.662 | 34.2 ± 130  33.1 ± 14.2 0.572 | 34.2 ± 12.0  30.8 ± 9.2 0.111 | NS |

| **Table S3 (cont.)** | | | | |
| --- | --- | --- | --- | --- |
| MUFA*,% energy*  Baseline  12-week | 19.1 ± 4.1  20.1 ± 4.2 0.217 | 19.9 ± 2.9  19.6 ± 3,8 0.566 | 19.4 ± 4.3  19.5 ± 3.8 0.869 | NS |
| MUFA, *grams*  Baseline  12-week | 53.3 ± 21.0  51.9 ± 21.2 0.641 | 53.8 ± 15.5  46.5 ± 13.3 0.003 | 53.0 ± 16.6  50.0 ± 14.3 0.270 | NS |
| PUFA, *% energy*  Baseline  12-week | 8.3 ± 3.6  8.9 ± 2.8 0.140 | 7.4 ± 2.5  7.6 ± 2.6 0.549 | 7.6 ± 2.3  8.1 ± 2.9 0.320 | NS |
| PUFA, *grams*  Baseline  12-week | 22.8 ± 9.9  22.1 ± 9.2 0.612 | 19.8 ± 7.6  18.9 ± 10.4 0.431 | 21.4 ± 9.4  23.3 ± 16.9 0.495 | NS |
| Fibre, *g/day^a^*  Baseline  12-week | 23.1 (16.1-30.2)  18.5 (13.3-23-.7) <0.001 | 21.0 (15.9-27.4)  17.0 (13.3-21.2) < 0.001 | 24.0 (19.4-28.6)  19.0 (15.6-27.2) 0.028 | NS |
| Alcohol, *g/day^a^*  Baseline  12-week | 1,69 (0.00-8.8)  3.60 (0.02-13.6) 0.068 | 4.51 (0,56-10.7)  6.43 (0.66-13.1) 0.279 | 5,97 (0.36-22.8)  6.30 (0.00-14.9) 0.589 | NS |

Data are expressed as the mean ± standard deviation: ^a^ median (25-75th percentile).

HC, carbohydrates; SFA, saturated fatty acids; MUFA, monounsaturated fatty acids; PUFA, polyunsaturated fatty acids.

Intra-treatment comparisons by Student’s t test and Wilcoxon test for related samples.

* P value for differences among treatments. ANOVA and Mann-Whitney tests. ^†^P for Enriched OJ versus OJ.
