# Supplementary material for: Effects of hesperidin in orange juice on blood and pulse pressures in mildly hypertensive individuals: a randomized controlled trial (Citrus study)
Source: Eur J Nutr. 2020 Jul 13;60(3):1277–88. doi: 10.1007/s00394-020-02279-0 (PMC7987641; doi:10.1007/s00394-020-02279-0)
Supplement: Supplementary file 2 — Supplementary file2 (PPT 159 kb) [file 394_2020_2279_MOESM2_ESM.ppt]

## Slide 1
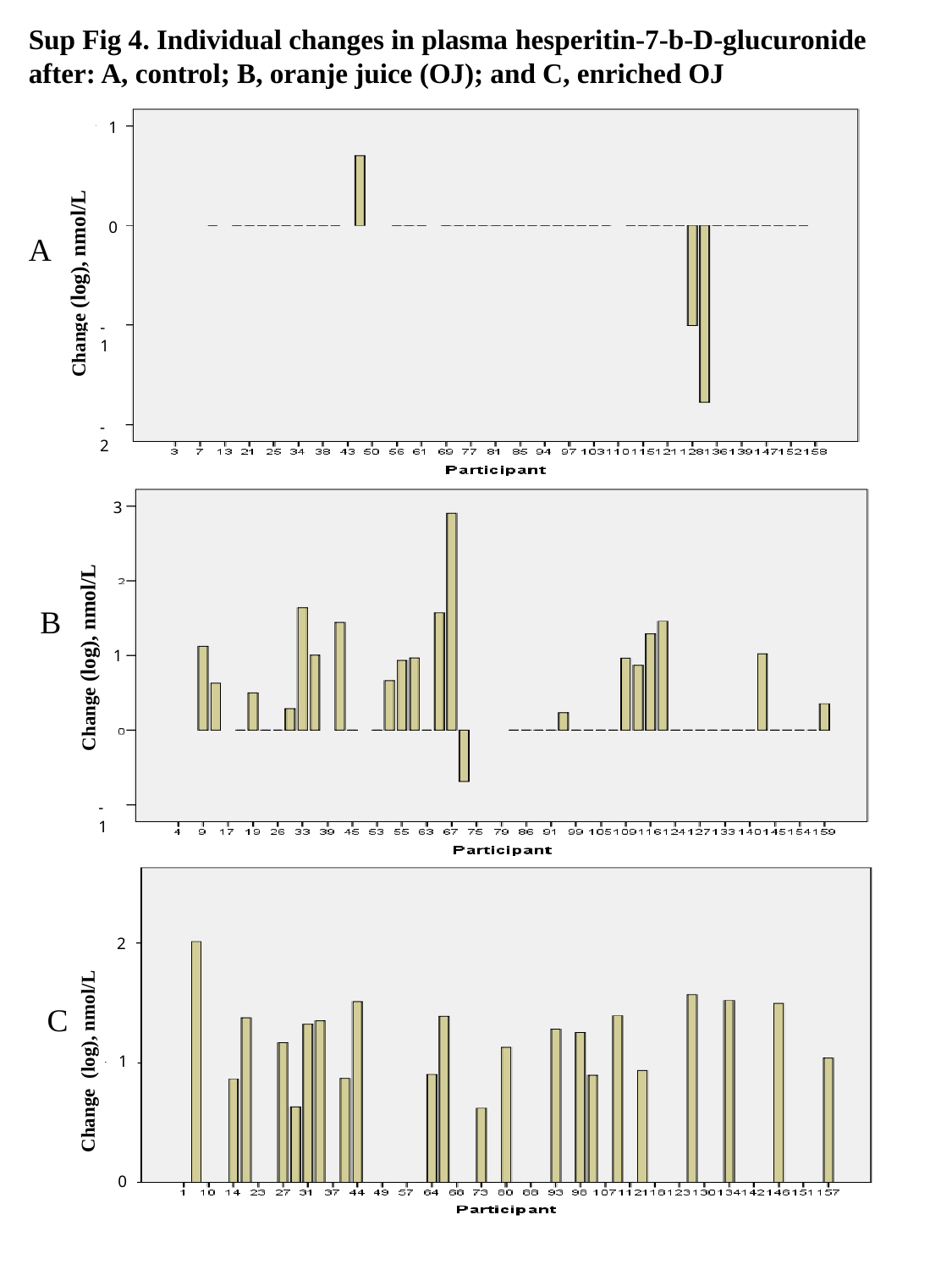

Sup Fig 4. Individual changes in plasma hesperitin-7-b-D-glucuronide after: A, control; B, oranje juice (OJ); and C, enriched OJ
1
0
Change (log), nmol/L
-1
-2
3
Change (log), nmol/L
1
-1
2
Change (log), nmol/L
1
0
A
A
B
2
B
0
C
C
1
