# Supplementary material for: Effects of hesperidin in orange juice on blood and pulse pressures in mildly hypertensive individuals: a randomized controlled trial (Citrus study)
Source: Eur J Nutr. 2020 Jul 13;60(3):1277–88. doi: 10.1007/s00394-020-02279-0 (PMC7987641; doi:10.1007/s00394-020-02279-0)
Supplement: Supplementary file 3 — Supplementary file3 (PPT 585 kb) [file 394_2020_2279_MOESM3_ESM.ppt]

## Slide 1
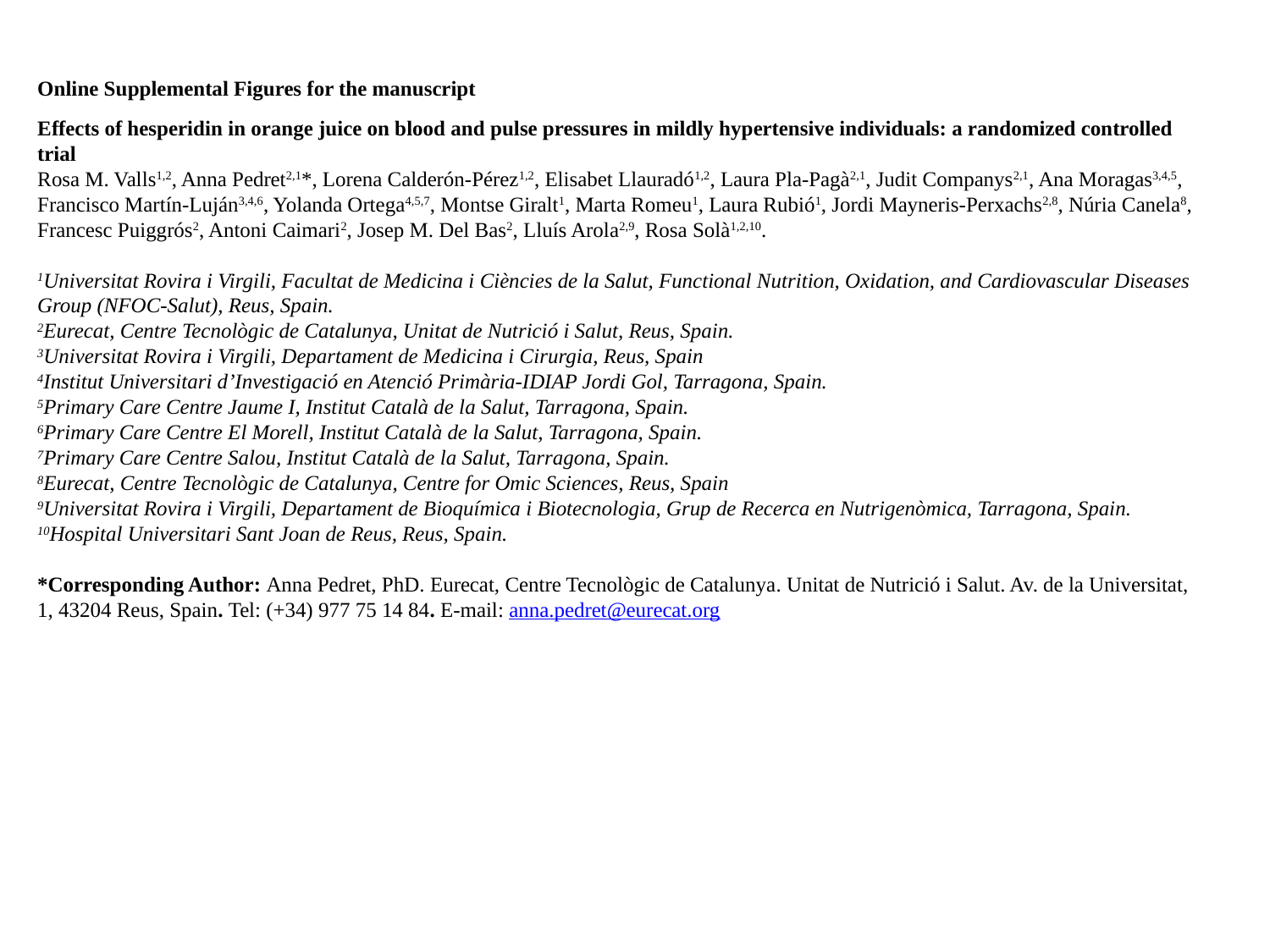

Online Supplemental Figures for the manuscript
Effects of hesperidin in orange juice on blood and pulse pressures in mildly hypertensive individuals: a randomized controlled trial
Rosa M. Valls1,2, Anna Pedret2,1*, Lorena Calderón-Pérez1,2, Elisabet Llauradó1,2, Laura Pla-Pagà2,1, Judit Companys2,1, Ana Moragas3,4,5, Francisco Martín-Luján3,4,6, Yolanda Ortega4,5,7, Montse Giralt1, Marta Romeu1, Laura Rubió1, Jordi Mayneris-Perxachs2,8, Núria Canela8, Francesc Puiggrós2, Antoni Caimari2, Josep M. Del Bas2, Lluís Arola2,9, Rosa Solà1,2,10.
1Universitat Rovira i Virgili, Facultat de Medicina i Ciències de la Salut, Functional Nutrition, Oxidation, and Cardiovascular Diseases Group (NFOC-Salut), Reus, Spain.
2Eurecat, Centre Tecnològic de Catalunya, Unitat de Nutrició i Salut, Reus, Spain.
3Universitat Rovira i Virgili, Departament de Medicina i Cirurgia, Reus, Spain
4Institut Universitari d’Investigació en Atenció Primària-IDIAP Jordi Gol, Tarragona, Spain.
5Primary Care Centre Jaume I, Institut Català de la Salut, Tarragona, Spain.
6Primary Care Centre El Morell, Institut Català de la Salut, Tarragona, Spain.
7Primary Care Centre Salou, Institut Català de la Salut, Tarragona, Spain.
8Eurecat, Centre Tecnològic de Catalunya, Centre for Omic Sciences, Reus, Spain
9Universitat Rovira i Virgili, Departament de Bioquímica i Biotecnologia, Grup de Recerca en Nutrigenòmica, Tarragona, Spain.
10Hospital Universitari Sant Joan de Reus, Reus, Spain.
*Corresponding Author: Anna Pedret, PhD. Eurecat, Centre Tecnològic de Catalunya. Unitat de Nutrició i Salut. Av. de la Universitat, 1, 43204 Reus, Spain. Tel: (+34) 977 75 14 84. E-mail: anna.pedret@eurecat.org

## Slide 2
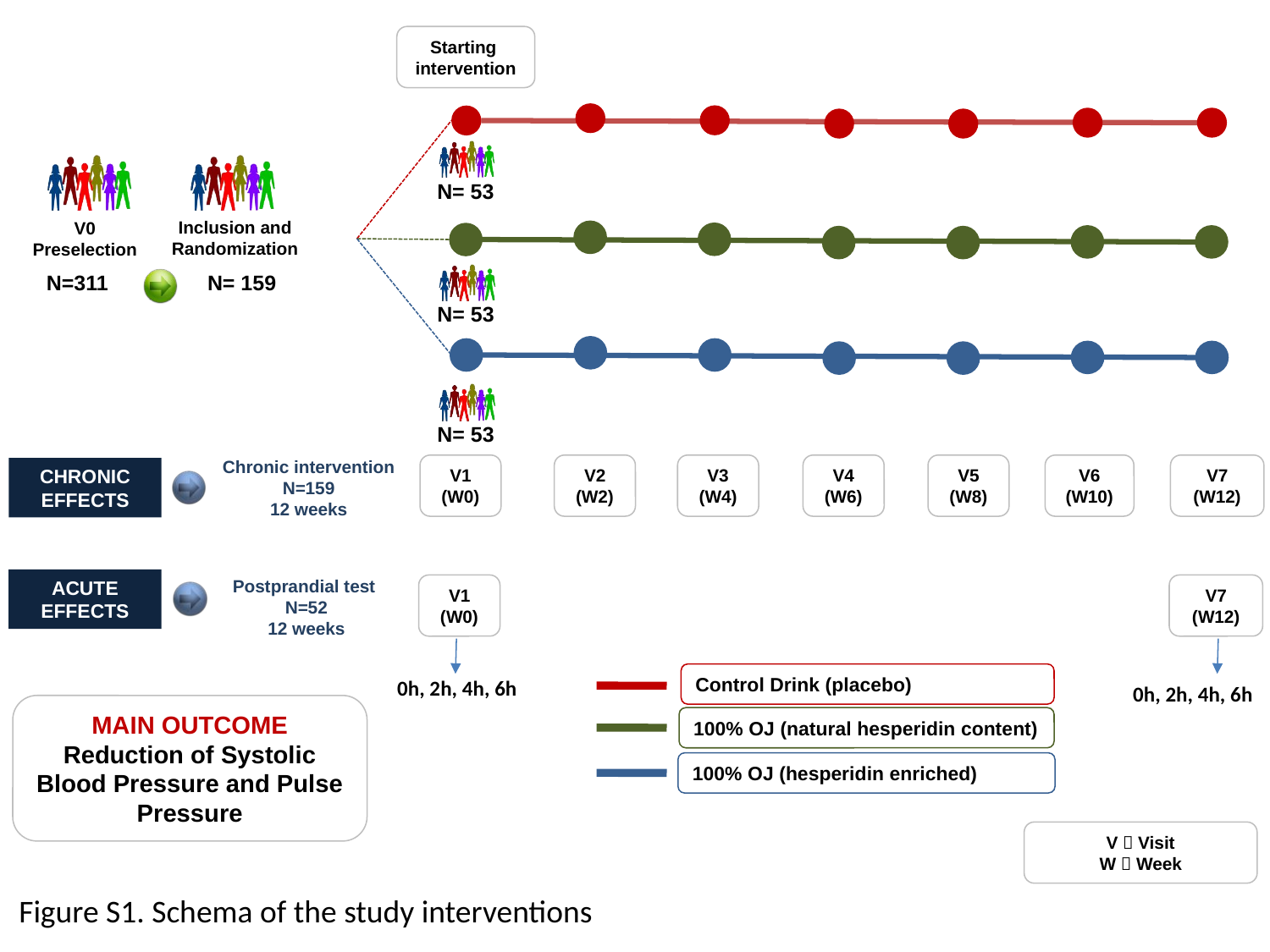

Starting intervention
V0
Preselection
Inclusion and Randomization
N= 53
N=311
N= 159
N= 53
N= 53
Chronic intervention N=159
12 weeks
V1
(W0)
V2
(W2)
V3
(W4)
V4
(W6)
V5
(W8)
V6
(W10)
V7
(W12)
CHRONIC EFFECTS
Postprandial test N=52
12 weeks
ACUTE
EFFECTS
V1
(W0)
V7
(W12)
Control Drink (placebo)
0h, 2h, 4h, 6h
0h, 2h, 4h, 6h
MAIN OUTCOME
Reduction of Systolic Blood Pressure and Pulse Pressure
100% OJ (natural hesperidin content)
100% OJ (hesperidin enriched)
V  Visit
W  Week
Figure S1. Schema of the study interventions

## Slide 3
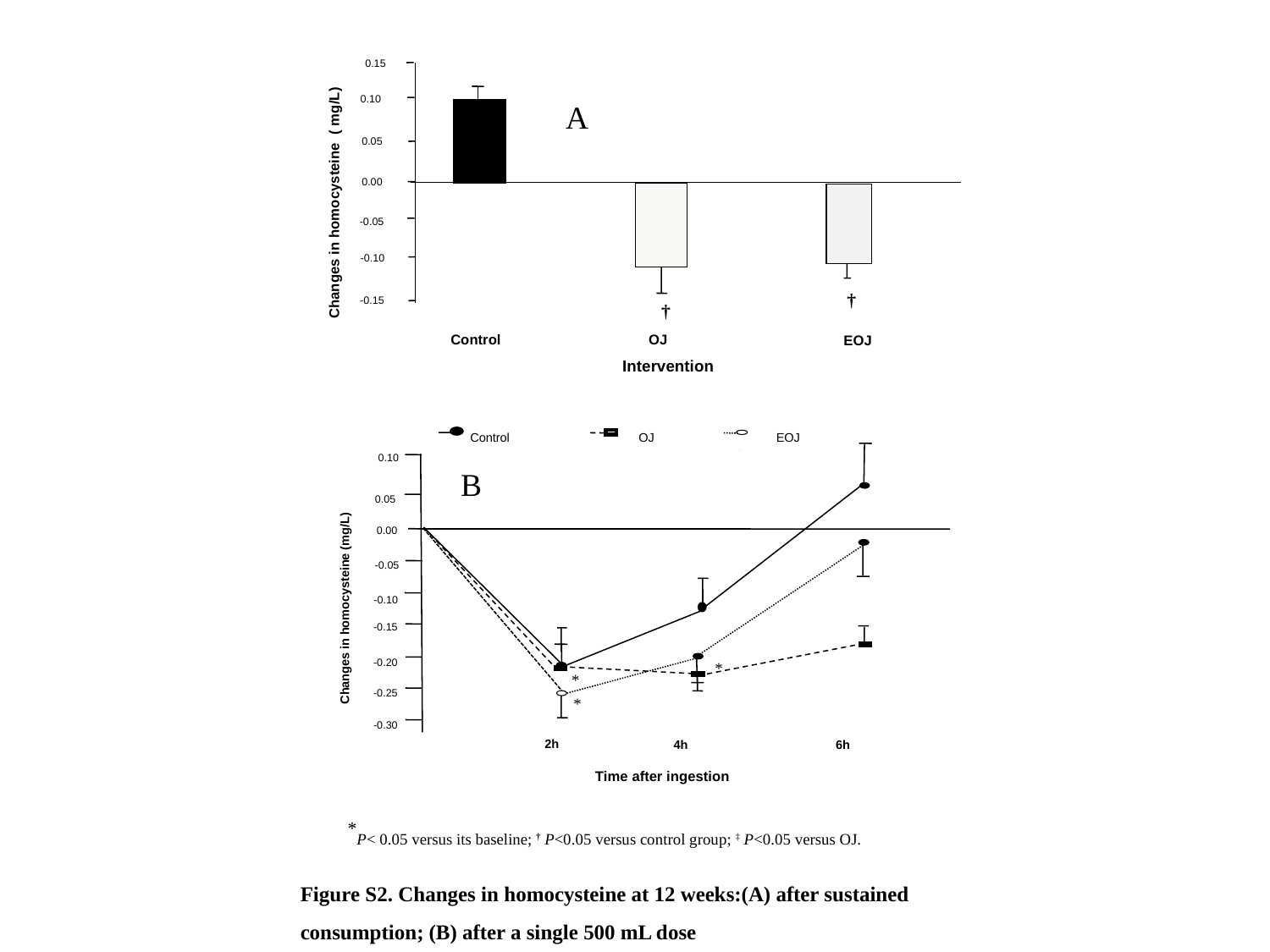

0.15
0.10
0.05
Changes in homocysteine ( mg/L)
0.00
-0.05
-0.10
†
†
-0.15
Control
OJ
EOJ
Intervention
Control
OJ
EOJ
 0.10
B
 0.05
 0.00
-0.05
Changes in homocysteine (mg/L)
-0.10
-0.15
-0.20
*
*
-0.25
*
-0.30
2h
4h
6h
Time after ingestion
A
*P< 0.05 versus its baseline; † P<0.05 versus control group; ‡ P<0.05 versus OJ.
Figure S2. Changes in homocysteine at 12 weeks:(A) after sustained consumption; (B) after a single 500 mL dose

## Slide 4
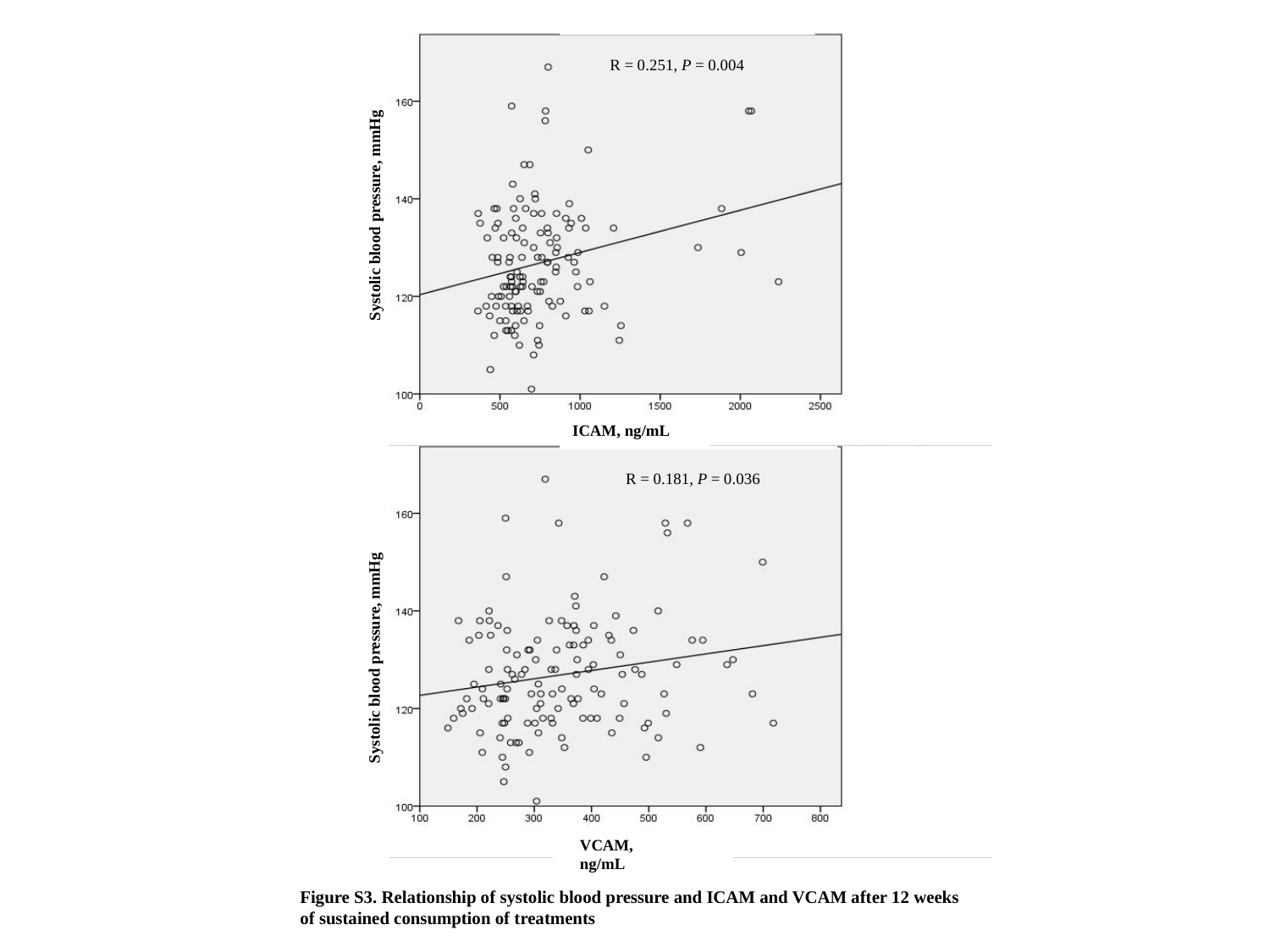

R = 0.251, P = 0.004
Systolic blood pressure, mmHg
ICAM, ng/mL
R = 0.181, P = 0.036
Systolic blood pressure, mmHg
VCAM, ng/mL
Figure S3. Relationship of systolic blood pressure and ICAM and VCAM after 12 weeks of sustained consumption of treatments
